# Cases Illustrative of the Efficacy of the Application of the Lunar Caustic in the Cure of Local Inflammation

**Published:** 1826-05

**Authors:** John Higginbottom

**Affiliations:** Member of the R. College of Surgeons of London.


ART. II.—*Cases illustrative of the Efficacy of the Application of the Lunar Caustic in the Cure of Local Inflammation.* By JOHN HIGGINBOTTOM, Member of the R. College of Surgeons of London.

IN my little Treatise on the Lunar Caustic, I have given, at page 126, an account of three cases of inflammation of the knee, successfully treated by the external application of that remedy. It is my present intention to call the attention of the profession to another case of local inflammation, in the cure of which the application of the lunar caustic has been speedily successful.

It must certainly appear striking at first that the caustic should subdue inflammation, and it may be extremely difficult to account for the fact. But it appears to me not the less certain that, to convert the cuticle of an inflamed part into an eschar, does, in certain cases, prove a mean of almost immediately destroying the inflammation.

Thus, in a fourth case of inflammation of the knee, I have succeeded in speedily removing the disease by the caustic, even without the application of leeches or poultice.

A servant girl, aged sixteen, applied to me with inflammation extending over the fore part of the knee, for which no cause could be assigned except kneeling in washing the floors. There were much pain and swelling, and the skin was exceedingly tender and hard to the touch; the pulse was frequent; and the patient was feverish, and complained of feeling unwell. I directed an emetic and purgative, and applied the lunar caustic all over, and a little beyond, the surface of the inflamed part. In two days afterwards, the swelling had much subsided, and the tenderness was entirely gone; some vesication existed on the inside of the knee, occasioned by the too free application of the caustic on that part; but over the fore part of the knee, where there was most inflammation, no vesication had taken place. The case gave no further trouble, but got well in a few days.

Similar cases, under ordinary treatment, have continued for many days, or even weeks, attended with much inflammation, and sometimes suppuration even has taken place.

This power of the lunar caustic in checking the action of inflammation, is not less obvious in the following cases, in which it will be observed that a tense and painful swelling of the hand was, in the space of forty-eight hours, rendered soft and puffy, and deprived of pain and of all the inflammatory action.

A servant maid, aged twenty-four, applied to me with a swelling of the middle finger, and of the back part and palm of the right hand, attended by such pain as to prevent her from sleeping in the night. She thought this affection had arisen from a puncture by a pin or needle, in washing. On examination, I perceived a small wound at the inside of the finger, at the first joint; and, on removing the skin by the lancet, a little pus escaped, and left a very small cavity. I applied the lunar caustic well within this cavity, and over and beyond the inflamed parts of the finger and hand, previously moistened with water, and left them exposed to dry. I directed an emetic and purgative medicine, and desired that the hand might be supported in a sling.

On the following day, my patient stated that her hand was perfectly easy, and had been free from pain from the time the sense of heat occasioned by the application of the caustic had subsided; she had passed a good night; the inflammation of the hand was completely checked in its progress; the swelling remained as before.

On the next day, the patient made no complaint; the swelling had become soft and puffy to the touch. In a few days more, the cuticle began to peel off; and in one point, where it was thick, there was a slight degree of tenderness. From this time there was no further trouble or complaint.

Another servant, aged twenty, slightly wounded the fore part of the index finger, at the first joint, by the bone of a hare, in dressing it. The wound healed in a day or two, and no notice was taken of it. In a few days afterwards, the finger became swelled and painful, and affected with diffused inflammation of an erysipelatous character, extending to the back of the hand, and there bordered by a ring of a more vivid colour. I applied the caustic nearly all over the finger, and upon the back of the hand, beyond the inflamed border.

On the following day, the swelling remained as before, and the patient complained of heat of the parts to which the caustic had been applied, and a small part of the finger, which had not been touched with the caustic, was very painful. I applied the caustic to this point.

On the succeeding day, the swelling had become puffy, and the whole finger and hand were free from pain. Several days afterwards, the hand was quite well. No medicine was given; nor was the patient prevented from pursuing her usual avocations.

This case was the more interesting to me, because I had had two similar cases a short time before, which had been occasioned by wounds received in cutting dog's meat, and which, under the ordinary treatment by lotions, had been several weeks in getting well.

The third case occurred in a country woman, without any assignable cause. There was much swelling of several fingers, and of the hand; one finger, and the back part of the hand and of the fore-arm, were much swollen, and the inside of the hand and two fingers in a less degree; and there was much pain. This affection had been coming on for several days, and had entirely prevented the use of the hand; there was some fever, and the tongue was white. An emetic, and a purgative with calomel, were prescribed; and I applied the lunar caustic over the whole surface, previously moistened with water, and left the whole exposed to dry.

Three days afterwards, the patient called upon me. The swelling had entirely subsided, and no pain had been experienced after the heat induced by the application of the caustic had ceased. It is worthy of remark, that a degree of pain felt at first in the axilla had also entirely disappeared.

I have already suggested, that to induce an eschar over the seat of an internal disease or inflammation, may prove of great service. I repeat the suggestion here, in order that it may excite the attention of some of the numerous readers of the London Medical and Physical Journal, and so be the more speedily submitted to experiment.

*Nottingham; March, 1826.*

